# Southern Dental Association—1894

**Published:** 1894-10

**Authors:** J. M. Walker


### THE DENTAL REGISTER.

Vol. XLVIII.] OCTOBER, 1894. [No. 10.

### Proceedings.

### Southern Dental Association.—1894.

Twenty-fifth Annual Meeting, Old Point Comfort, Va., Augnst 2-6, 1894.

Reported for the Dental Register by Mrs. J. M. Walker.

[*Continued from September Number, page* 450.]

At the first session a resolution was adopted exempting all charter members from further payment of dues.

Also a resolution reinstating members liable to loss of membership for non-payment of dues, on payment of arrears for two years—$6.00.

The following new members were elected: J. W. Boozer, Columbia, S. C.; S. H. McKee, Americus, Ga.; J. E. Frazier, Birmingham, Ala.; C. V. Rosser, Atlanta, Ga.; I. N. Carr, Tarboro, N. C.; John S. Marshall, Chicago, Ill.

The committee on investigation of charges against Dr. E. B. Marshall, Rome, Ga., not having found any evidence of continued violation of the code of ethics, his advertising cards having been promptly withdrawn from publication, on motion of Dr. Beach, seconded by Dr. R. R. Freeman, the charges were ordered dropped in view of his long services as a charter member of the association, and his frank letter to the committee expressing regrets for his past errors due to misinterpretation but not to a knowing violation of the code. The committee was discharged.

PROSTHETIC DENTISTRY.

Dr. Thos. P. Hinman, Atlanta, Ga., read a paper entitled: "An Original Method of Protecting the Tips of Porcelain Teeth in Bridgework," also "A Simple Method of Banding a Logan Crown."

He said: The most beautiful piece of bridgework is no stronger than the fragile porcelain facing that gives it its beauty; how then shall we best protect their frail portions?

In the first place always use "up-and-down" pins rather than "cross-pin teeth." The strain is more equally divided as the pins are in the long axis of the tooth and further apart. To protect them from the strain of mastication, bevel the tooth on the lingual side, from the lowest pin to the cutting edge, to a knife edge, using fine corundum stones. Back with 22 k. 29 g. gold, but not bringing the backing over the bevel, cover the backing with base plate, wax and invert. Remove the wax and flow solder to restore contour, except on the beveled portion. After the bridge is completed, burnish the remaining portion of the backing not covered with solder to the beveled portion of the facing, thus protecting the porcelain from the strain of mastication by making it on an inclined plain. If the gold is burnished down over the beveled portion before the solder is flowed, the contraction of the metal will break the porcelain.

To band a Logan crown, prepare the tooth as for a Richmond crown. Make a band of 22 k. 29 g. gold extra wide and larger at the opening than at the root end. Remove the band and grind the crown to fit the root approximately. Replace the band and drive the crown home with the band hugging tightly. Remove the band and crown and with corundum stone grind away the band in front leaving it very long in front. Grinding towards the tooth will burnish the band to it; burnishing the longest portion of the band down over the bulbous portion of the tooth at the back makes a perfect joint with no inside disk and no soldering; is strong and durable.

Dr. John S. Thompson, Chairman of Committee on Prosthetic Dentistry, reported no other papers ready from this committee, Dr. Hinman's paper was opened to discussion.

Dr. H. A. Parr, New York, having been requested to open the discussion, said that he knew nothing about Logan crowns, never having used one, but that there was nothing new in the method described of backing a tooth. The trouble with porcelain teeth backing is due to the unequal distribution of heat in soldering.

Dr. Hinman replied that the only original feature to which he laid claim was that of burnishing the backing to the beveled portion of the porcelain after the piece is soldered.

Dr. W. H. Morgan said it was certainly a new idea to him that gold could be brought into perfect contact with the tooth and stay there. His experience was that it would always spring back.

Dr. Parr: Every time you touch the gold you put more spring in it. When you get it where you want it let it alone. If pure gold is laid on loosely and properly invested, the solder will bring it to a close joint and the porcelain will not break unless a very heavy strain is put upon it. We may not make a perfect joint so there will be no leak between metal and porcelain, but we do the best we can.

Dr. W. C. Barrett, Buffalo, N. Y., agreed with Drs. Parr and Morgan as to the impossibility of burnishing gold to a close joint with porcelain. If, however, it could be demonstrated that this could be done, it would be a great step in advance, and would repay him for coming to this meeting. He would always flow solder to the point of the tooth. He spoke of the great advantages offered in crown and bridgework in saving teeth formerly condemned to the forceps, and in this respect was doing much to elevate prosthetic dentistry above the low level of many years.

Dr. L. P. Dotterer, Charleston, S. C., described a method of restoring a bicuspid in which the buccal portion was entirely gone, the palatine wall standing and the patient unwilling to have this cut off or to wear an "artificial tooth." He banded the lingual portion of the tooth, adjusted a backing to a central incisor face, soldering the sides of the backing to the gold band, and strengthening with solder to form the grinding surface, thus making a hollow crown to accommodate the palatine portion of the natural tooth, the back and grinding surface being all gold, with enough porcelain on the buccal portion for effect.

Dr. H. E. Beach did not think that pure gold merely burnished to the beveled portion of the tooth, as is Dr. Hinman's method, would afford sufficient strength; it is too soft and malleable and requires solder flowed over it to stiffen and strengthen it and protect the frail edge of porcelain, allowing, however, only a slight line of gold to show at the cutting edge.

Dr. R. R. Freeman gave his method of securing perfect occlusion of molars and bicuspids in bridgework. Get the bite in modeling compound and flow Melotte's fusible metal in this, giving an exact model on which to adjust the work without wearing away the model as when it is made of plaster of Paris.

The occlusion can be swaged between two metallic casts and made very perfect. In heating the fusible metal put a little water in the spoon with the metal, you will thus get a heat of 212° and the metal sets immediately.

A member spoke of the advantages of the English tube teeth in bridgework, they are the least unsightly, requiring no gold tips, and are easily replaced if broken off. He has been using them for several years and the more he uses them the more fully he appreciates them. He regretted there was no blackboard that he might illustrate their use both for bridgework and as pivot teeth.

Dr. W. G. Browne places a compressed gold cylinder under a backing of very thin platinum, and burnishes only approximately to the surface of the porcelain; when ground to a feather edge the bevel brings it perfectly in contact, and the solder follows the compressed cylinder and fills in very thoroughly.

Dr. S. B. Cook does not attempt to burnish down, but simply presses it to place with a soft piece of wood, annealing as necessary. He thinks the porcelain breaks because the enameled surface is broken in grinding, and that this is as injurious to a porcelain tooth as to a natural tooth; it is like taking off the dense surface of vulcanized rubber.

Dr. Parr seldom uses a porcelain tooth that he does not grind off some portion of the surface to get a perfect articulation and does not believe that it renders them any more liable to break.

Dr. J. Y. Crawford thinks this tipping and shoeing of porcelain teeth a useless expenditure of time and labor, breaking more teeth than it preserves. We must rely on non-occlusion of the porcelain, making the point of contact on the strong backing reinforced with solder. He backs the palatine surface only, making the gold slightly concave. Covering a backing of 22 k. gold entirely with 22 k. solder, piling on enough to give all the requisite strength, making it as near the size and shape of the natural tooth as possible. Before soldering the gold should be pickled to virgin purity, blackening and discoloration of the gold resulting from charred impurities.

Dr. W. H. Morgan: The idea that cutting the enamel of a porcelain tooth weakens it is exactly the reverse of the fact; the strength of the tooth lies in the body, not in the enamel. In soldering there must be a foundation for the solder to take hold of; instead of burnishing, better fray the surface with a rough file, and give the solder something to take hold of. Attempting to burnish the gold backing to the tooth is like planting your elbow in a feather pillow, it will cock up at the ends. Burnishing the gold hardens that side and makes it contract and draw up.

Dr. Parr: You have to anneal the gold and platinum the same to take the spring out of it. All these little ideas are not *new* ideas. They were known years ago to the old men who laugh at these *new ideas* of the young men.

Dr. Hinman being called on to close the discussion of his paper, said that he did not regret having read it as it had called forth a discussion from which he had learned "much." He still believes, however, that the gold used in the way he describes does hug the porcelain and make a close joint, not with the burnisher but with sand paper and cuttle-fish finishing.

Dr. Crossland: I thought I burnished the gold close to the tooth, but if all these old and experienced men say it can't be done, I suppose I don't, but it looks like it!

The subject of Prosthetic Dentistry was not reached again until the last session, Monday morning, when

Dr. H. E. Belden, New Orleans, La., read a paper entitled: "Articulators." After enumerating the essential features of articulators in general and the advantages and disadvantages of those now in use, he named as the special features of the Belden Articulator, that the bite can be lengthened or shortened while the plates are kept parallel; that it is capable of extension between plates for full cases, or of being brought in close apposition in single cases; that the upper plate slips back and forth to accommodate individual requirements; that the bite moves from one side to the other to correct the patient's bite; that it has a hinge-like motion to try the occlusion of each tooth as it is waxed on; that it allows a complete view of the palatine portion while the plates are closed, thus showing the articulation of the inside cusps, preventing tilting, rocking or fracturing the plate; that there are no small screws or detachable parts to be lost in the debris of the laboratory; finally that it is light and compact, and commends itself to all as a true and reliable articulator.

Dr. Noble, Chairman of Committee on Voluntary Essays, read a paper from Dr. L. G. Noel, Nashville, Tenn., entitled: "Two Useful Ideas in Prosthetic Dentistry."

The first is a combination of gold and vulcanite for bridges. Make and place in position gold shells for the teeth that serve as abutments; bring them away in an impression, from which make a model in sand and plaster. Connect the shells with a strong bar of platinum and iridium to give horizontal strength and afford attachment for the rubber. Place moulding composition about the bar and adapt it to the gum while soft, directing the patient to close the teeth upon it for a bite. Adjust suitable vulcanite teeth, and finish by usual process. It is often not necessary to take a bite, but simply wax the teeth to the bar and arrange in the mouth for articulation. The rubber can also be cut away so as not to rest on the gums so that it will not be necesary to get an impression or make a model of the gum. There are no crevices for the collection of fluids and food; it offers greater resistance to the strain of mastication than soldered teeth. There are many modifications of this idea possible; two bars can be soldered in and swaged gold grinding faces vulcanized in; fill in with solder, catching some platina pins in solder for attachments; wax on and bite into adjustment. If the teeth for abutments have long crowns and small necks extend the gold shell only to knuckle and thus avoid cutting away the enamel to get straight lines, removing the grinding surface to admit the thickness of gold in articulation. Extend the end of the bar past the shells for posterior teeth. In a case of inserting the two superior laterals, collars were cemented to the canines connected by a bar across the lingual surface of the centrals, with the laterals backed and soldered to the bar. A bar thus attached can be utilized in cases of regulating.

Dr. Noble also read a paper from Dr. G. N. Johnson, Concord, N. H., entitled: "Unprofessional Prosthetics," (especially the prosthesis of rubber plates.)

Dr. Johnson deplored the relegation of prosthesis among the musty relics of the past, the fashion in dentistry now is all on the line of theory and scientific investigation. Prosthesis was almost entirely ignored at the Columbian Congress while much time was given to hypnotic suggestions. A high-toned dentist airily claims that his practice is "chiefly operative," admission of prosthetic work apparently dimming the light of his professional halo. College graduates are often not able to construct a passably good set of teeth. Examining boards dwell long on the treatment of exposed pulps and septic canals, while the most abject ignorance on the subject of artificial dentures is no barrier to passing the applicant. But there is no branch of dentistry which requires more skill, judgment, scientific information than the adaptation, expression and articulation of a denture. Dr. Johnson then reviewed the symptoms of what is known as "rubber disease," and claims that the true etiology is found in the rough surfaces, knife-like edges, suction ridges, the cess-pools walled in to increase their capacity for filth, known as "air chambers." All the sharp irregular ridges, undercuts, overhanging edges, etc., are carefully preserved and presented to the delicate membranes of the mouth, presenting a surface better fitted for a nutmeg grater or a beef-steak crusher. After a little absorption, a little recision, a slight change of bearing by wearing down of the opposing teeth, and a new resting place has to be found for all these excrescences. Whether prosthetic dentistry is professional or otherwise depends, not on the material used, but on the ethics which govern and the science which directs its use. It is our professional duty to render careful, intelligent and conscientious service in any direction it may be called for.

These papers having encroached upon the time set apart for the annual elections, they were passed without any discussion.

At an earlier session, in connection with the papers on hygiene, Dr. John A. Daly read a paper entitled: "Hygiene in Prosthetic Dentistry."

He said—The care of the human mouth made dentists the pioneers in the discovery of the importance of the bacterial theory of disease which clothed the profession with new duties and larger responsibilities as sentinels at the gateway of public health. The theory has possibly been pushed too fast, if not too far, but we stand on solid ground when we say that prevention is the greatest function of the healing art. Cleanliness in things physical yields nothing to moral purity in the canons of right life; it is a useful handmaid to preventive science. The structure and functions of the mouth offer serious difficulties to the maintenance of that hygienic status which sanitation demands. If a man could be scolded or flattered into taking proper care of himself, the history of medicine and surgery would have to be written anew from the beginning; there would have to be a new birth of the present dental and medical practitioners.

Dentistry in connection with this subject has many burning questions to solve—many professional sores to salve. In the fore-front is the question of vulcanite dentures; twenty years ago we were told that we were in honor and duty bound to dismiss it utterly and forever, and yet it holds its place to-day because there is nothing to take its place. The evils charged to it are all preventable evils. The vulcanite plate as usually made presents a rough, uneven surface; if most skillfully made it is porous, if unskillfully made porosity is increased in proportion. This spongy structure, if we accept the bacterial theory, is a microbic hive or nursery, providing there is the warmth and moisture necessary for their generation, growth, propagation and nourishment. Again, being porous, it is soon saturated with the fluid secretions of the mouth, charged with organic matter in a state of decomposition which is disgusting and alarming.

A regard for common decency and the ordinary safeguards of health should not tolerate, much less participate in, creating methods leading to such menacing consequences. It has been claimed that these consequences are avoidable by ordinary habits of cleanliness on the part of the wearer, but this is not so. The wearers are not provided with the chemical solvents in which it would be necessary to steep it.

But simpler means are neither undiscoverable nor unattainable. The cheap, durable vulcanite plate can still be retained and kept in hygienic condition by interposing an efficient and suitable metallic lining between the palate and the plate. This is a thin, pliable plate of pure gold, smooth on the palatine surface, and incorporated with the vulcanite with absolute mechanical union.

A sheet of No. 60 gold foil, smooth on one side and with a rough, crystalline surface on the other, is made to fit closely into plaster molds, making a plate of vulcanized rubber on a golden base, so that they cannot be separated by mechanical means, except heat or such chemical disintegration as would destroy the denture itself; a dense, lustrous, pure gold surface, which imparts beauty to the plate—an added element of value. The lingual surface of such a plate has the highest density and is susceptible of an attractive polish. The plate is an accurate copy of the model and is practically perfect. It will occasion no rubber sore mouth, harbors no disease germs or poisonous secretions; decaying accumulations, uncleanliness, unsightliness and malefic odors are reduced to the minimum. This lining is applied in less time than is required to clean the dirt and plaster from an unlined plate. The common vulcanized denture lies under strong suspicions of being an active agent in producing serious attacks of sore throats, neuralgia and indigestion; that they aggravate these disorders is axiomatic, and no physician will permit a patient thus affected to wear an ordinary vulcanite plate until restored to full health and vigor. Gentlemen: we need to look *higher than the tongue* when we diagnose disease or ill health.

The paper was passed without discussion.

### OPERATIVE DENTISTRY.

When this section was called, Dr. E. P. Beadles, Danville, Virginia, read a paper entitled: "Anterior Fillings," of which a brief synopsis follows.

The paper was limited to the four upper incisors and the two cuspids, with reference to the operator himself, the preparation of the cavity, and the introduction of the filling material. The proper position for the operator was first considered. No operator, no matter how expert, can perform good work in a cramped, awkward or fatiguing position. To be the least tiresome, his position must be a perfectly natural one; the back bent, or the head twisted to one side will soon bring fatigue and the best results will not be attained. Dr. Beadles considers the proper positions for the operator, in operating upon the anterior teeth (and with slight modifications it is true for all the teeth), to be an upright position, standing to the side and partially behind the patient, with the head bent slightly forward. To this position the mouth mirror is an indispensable adjunct. In proof of the correctness of this method Dr. Beadles says: "I never had a backache in my life."

Dr. Beadles was especially emphatic in commending the constant use of the mouth mirror as an adjunct to the comfort of both operator and patient. In the preparation of the cavity, he said: Do not mutilate the teeth; nature knows best, and no man yet has improved upon her forms. Do not use rubber wedges for separating; dont use separators with too long a lever. All the space needed is room enough to polish the filling after its insertion, and if you have not this, wedge in a small piece of dry cotton, not too tight, and dismiss the patient until the next day, when there will be ample room. Cut away all the enamel wall on the lingual side, exposing the entire cavity to the eye as reflected in the mirror. The shape of the cavity when ready for the reception of the gold should be that of a cube or square with rounded corners; sometimes a slight groove is required in the cervical wall for the reception of the first piece of gold which should be non-cohesive always. The sides should be as nearly parallel as possible, no undercuts, no pits or retaining points. Fill over-full the groove mentioned, condense well over the cervical margin and burnish then and there. By doing this you have the margin safe and need not interfere with it again. From this point on, Dr. Beadles generally uses cohesive gold, rolling the gold between the fingers, annealing and cutting into pellets the desired size. Keep the proximal wall well burnished as you proceed, and you have finally only to polish with fine sand-paper disk finishing with use of cuttle-fish.

For instruments Dr. Beadles requires only one small, straight, round-pointed, steel-handled plugger, one flat-pointed and one or two right-angled, round-pointed, ebony-handled pluggers. When packing against the wall use smooth points, though serrated points may be used in the body of the filling if desired. Don't pound and hammer the gold too much, which only weakens it, and when the cavity is filled *stop*. Don't build on gold simply to grind it off again. Using hand-pressure, very frail walls can be built against with safety. Dr. Beadles affirmed his strong faith in hand-pressure. More force can be used as the pressure is steady and gradual, and no mechanical invention can improve upon the human hand. In many cases a cavity in the anterior teeth can be nearly filled with cement, pressing annealed gold into the soft cement, waiting for the cement to harden before finishing the filling. Amalgam may also be found desirable in a few cases, facing the labial surface with gold.

Dr. T. W. Foster, Decatur, Ala., next read a paper entitled: "Some of the Merits of a Contour Filling Made in the Laboratory."

No matter what the operation may be, it is the duty of the operator to consider the welfare of the patient, not only in the use of the best material and his highest manipulative skill, but also to adopt the method calculated to give the least pain and discomfort to the patient. Failure in this respect makes the dental profession responsible for the universal apprehension experienced in contemplating a visit to the dentist. In view of this fact Dr. Foster wished to call attention to the merits of fillings *made in the laboratory* for large contour work. The case described in the paper was illustrated by a diagram showing the mesial corners of the superior central incisors broken off from the gingival margin of the mesial surface diagonally across to the angle of the distal surface. The patient was a machinist and the teeth had been thus broken off by a blow from the handle of an iron maul. As a rule, in such cases, Dr. Foster would remove the pulp and the remaining portions of the crown and replace with Richmond or Logan crowns, but the patient, as is often the case, revolted at the idea of "artificial teeth," and would have nothing but gold fillings. To satisfy him, the right central was contoured in the usual way, the operation consuming three hours, both the patient and himself being worn out at the end of that time. In order to make it easier for both parties the left central was restored by a filling made in the laboratory, as follows: The enamel margins were beveled accurately and thin platinum burnished to the surface extending beyond the margin of the fracture. Through the platinum three holes were drilled at parallel angles in which were inserted platinum pins. The tooth was then contoured with wax from the labial surface, another piece of platinum was burnished adhering to the wax. The whole was then removed and invested, leaving the mesial surface exposed. The wax was burned out and the pocket filled with 22 k. gold solder, the investment removed, the piece finished and adjusted in little over an hour. The patient was delighted with the operation, so very easy for him and with the result also, and wanted to know why in the world he had not done the other tooth in the same way. Just before leaving home, Dr. Foster had examined the two fillings for comparison, and found that while the filling contoured at the chair was slightly pitted on the surface and the angle of the mesial corner slightly defaced, the "laboratory contour" was as perfect in every way as when it was inserted and had a much better *expression* than the "foil contour." While the final result is thus in every way satisfactory, the ease and facility of the work for both operator and patient commends itself to the consideration of all. For æsthetic results a porcelain-faced filling, as described by Evans, would, of course, be preferable, but much more difficult.

In reply to a question as to how the laboratory made filling was attached to the tooth, Dr. Foster replied: "With Justi's cement."

The papers of Drs. Beadles and Foster were opened for discussion.

Dr. Frank Abbott said that while many operations in the mouth were more or less painful, it was the duty of the operator to inflict as little pain as possible consistent with the proper performance of the operation. They are so easily rendered excessively painful by careless operating, but in order to encourage people to have their teeth cared for, as little pain as possible should be inflicted. It is exceedingly difficult in many cases to adjust the rubber dam without inflicting severe pain, and for this reason Dr. Abbott has abandoned its use except in extreme cases. It takes so much time and gives so much trouble and causes so much pain that its use is superseded in his practice by other means that occasion less pain, give less trouble and consume less time. Time wasted in accomplishing something unnecessary can never be made up again. In the use of obtunding material, twenty-five or thirty years ago, arsenic was almost universally applied in cases of sensitive dentine, and the result was numerous dead pulps, abscesses, etc. Now the country is flooded with all kinds of nostrums from all parts of the country. The *alkalies* are nature's natural obtundent.

The teeth are not naturally sensitive, but when inflamed under the action of the acids which cause decay, they become excessively sensitive. An alkali counteracting the acid restores the tissues to normal condition. Soda answers every demand and is usually harmless, but the patient must be put under regular treatment for some time, as follows: A teaspoonful of soda in a glass of water, using a little to rinse the mouth and hold in contact with the teeth twenty or thirty times a day. The second day the proportion of soda may be doubled. Then the soda may be placed directly in the cavities, and by the fourth day the teeth can usually be cut without pain. Very much of the pain attributed to dental operations is caused by bungling operators. There is everything in knowing just what you want to do and how to do it.

The question of the sensitiveness or non-sensitiveness of normal dentine was discussed at great length with the final conclusion, as stated by Dr. R. R. Freeman and Dr. Beech, that it is a question which can never be solved except theoretically, absolutely normal dentine being inaccessible. When exposed to tests through removal of its covering—the enamel—it is no longer normal.

Dr. Crawford criticized the use of platinum in contact with the dentine in Dr. Foster's "laboratory contour." He would use pure gold adapted to the walls with soft-wood points. Dr. Crawford objects seriously to the use of any two metals in a tooth, as platinum and gold in Dr. Foster's case, and especially the combination of metals in the amalgam alloys.

Dr. W. H. Morgan has had no experience with contour fillings made in the laboratory, but has found contour work done at the chair so satisfactory that he sees no means for changing his methods. He does not understand how or why such a filling as that in the right central could require over three hours' time or prove exhausting to either patient or operator. On one occasion he had a similar case except that it was necessary to fill the root also. Being a clinic "the boys held the watch on him," though he was not aware of the fact. The filling consumed six and a half sheets of No. 5 gold and was completed in fifty-five minutes by the watch.

Dr. Foster: I did the best I could and I learned under you! Dr. Morgan objected to the dogmatic style of Dr. Beadles' paper as not being scientific. No set rules can be laid down to fit every case and every operator. He said: I have been filling teeth for many years, but I do not always use the mouth mirror. I have other use for my hands in many cases. Standing "perfectly erect at the side of and partly behind the patient" will not answer in every case. I want to look into the mouth and see what I am doing. I want to see the other end of my instrument, and there are many cases where I prefer to stand in front of the patient, and often at the left side. In regard to the rubber dam causing so much pain that it is better not to use it at all, I find that I inflict as little pain and inconvenience with as little trouble and time with the rubber dam as with anything that can be substituted for it. Neither can I endorse the rule of never using rubber wedges. There are cases where nothing else will do, though I always seek to inflict the minimum amount of pain. In some cases enough cotton cannot be inserted without crowding it up into the gum in the interdental space.

Neither is hand-pressure the one mode above all others. The true philosophy in these matters is to examine into all the conditions and be governed by the environments and not say, dogmati cally, this way only and never any other. Much depends on the idiosyncrasies of the patient and every case must stand on its own merits. Dr. Morgan spoke of the various methods of obtunding sensitive dentine and commends the spray apparatus with ether, cooling the tooth until it is benumbed, but not continuing it too long, which causes excessive pain. The tooth is less sensitive if kept perfectly dry, and this is impossible without the use of the rubber dam, which, therefore, becomes a very important adjunct in lessening the pain caused by cutting the dentine. In regard to the general use of arsenic, as mentioned by Dr. Abbott, Dr. Morgan thinks it could only have been used by the ignorant class of practitioners, as even horse-doctors have always been familiar with the effects of arsenic in causing slough of soft tissues, as used by them in curing fistula, etc. A very little particle of arsenic inserted in the fistula and closed in, has sometimes caused diseased tissue "as big as your head" to slough out. Any dentist, except the very ignorant, must have known it would cause the death of the soft tissue in the tooth and beyond.

Dr. Friedrichs uses hand-pressure in preference to mechanical. The rapid blow of the latter hardens and condenses only the surface of the gold, while hand-pressure spreads it in all directions. His experience has been that the mallet is very objectionable to the patient, and unfavorable to the operation.

Dr. Cowardin thinks that the sensitiveness of dentine is due largely to individual idiosyncrasy and constitutional conditions. Patients of rheumatic and gouty diathesis suffer exquisitely from sensitive dentine. The dentine is abnormally sensitive from constitutional irritation.

Dr. Frank Holland does not see what the condition of the blood has to do with the condition of the dentine. If a patient is irritable from disease he will complain more, but that is no proof that he suffers more than another.

Dr. Barrett: Pain is the index to abnormal conditions. Normal tissues are not sensitive. There is no true nerve tissue in the dentine. The protoplasmic elements contain the elements of nerve and other tissues but normal dentine is utterly and entirely without sensation. The pulp itself in its normal condition is without sensation. How often do you not, in excavating a cavity, expose and touch the pulp and the patient is not cognizant of it, but if you allow it to remain exposed until it becomes inflamed, then it becomes exceedingly sensitive. Only when abnormal through irritation does it awaken to the power of conveying the sensation of pain.

Dr. Friedrichs: Is that a hypothesis, or can it be demonstrated?

Dr. Barrett: It is simply axiomatic and does not need demonstrating any more than that 2+2=4.

Dr. Pierce does not agree with Dr. Barrett. Whether normal or not normal, terminal filaments are always sensitive. You cannot touch them without thus conveying an impression, though the degree of pain may vary. In the interzonal layer you will always get a response, at the border line passing from the enamel to the dentine.

Dr. R. R. Freeman to avoid the continued pain, day after day, caused by the use of separating rubber, wooden wedges, cotton, etc., uses the modern mechanical appliances known as *separators* with wonderful satisfaction both to himself and his patients. He said—Put on the separator and start the screws, look your patient in the eye and presently you will see a look come over the face that warns you to stop; let down a little and wait, the patient will conclude "why that wasn't as bad as I thought;" then start it again, and you will easily get all the space you want, and it is all over at one sitting without prolonging the agony for days and perhaps a week. I began to use the rubber dam as soon as it was invented because I knew I would give less pain if I could keep the cavity dry, and it gives me the free use of both hands so that I can work to better advantage in every way. There are some irregular cases where these appliances will not fit and where neither rubber nor wood will answer, and then you must resort to cotton. Where it seems impossible to get anything in, that is where cotton works to the greatest advantage, roll a little particle of cotton fibers in a waxed silk ligament and you can draw it in between the teeth, tying it so that it will not work up to the gum, and the moisture will swell that little bit of cotton and give you the needed space. In some cases pain is a benign influence and is wanted as a warning, but there is no necessity for inflicting avoidable pain. Establish the confidence of your patients that you are not going to inflict unnecessary pain; make them feel that you are the captain who is going to carry them safely through, *and they won't suffer!*

Dr. Beach endorses Dr. Freeman's use of the separators. He does not believe in the slow process of wedging, with its prolonged pain. He knew from his own experience that even a little fiber of floss silk broken off between the teeth causes worry and irritation till he gets it out, and therefore he will not subject his patients to wedges of rubber, wood or cotton to remain between the teeth for several days. He spoke of his own experience with a duct-compressor, which theoretically promised to be something very nice. After having it used in his own mouth, he would never consent to use it on one of his patients though it might be very lovely as far as his own convenience was concerned.

Dr. Beadles, in closing the discussion, said that he had not intended to lay down any dogmatic rules for the governance of others. He had only desired to state the methods through which he had personally attained the greatest success, that possibly some might be benefited by the hints thus thrown out.

The subject of Operative Dentistry was passed at the close of this discussion, but at a subsequent session several voluntary essays were read which properly belong to this section.

Dr. W. V. B. Ames, Chicago, read a paper on a new cement which he calls "Oxyphosphate of Copper" and which he considers invaluable in what may be called "desperate cases." It is compounded by mixing black oxide of copper-cupric oxide with a solution of phosphoric acid. A phosphate of copper *per se* is formed which is held in solution in an excess of phosphoric acid. The cement is used in a plastic state and penetrates the tubuli of the dentine exerting a positive embalming effect. It is insoluble in the fluids of the mouth after crystallization. It is contra-indicated for crown setting from its caustic nature, but is valuable for filling purposes where conditions admit. It cannot be used indiscriminately as it might cause an occasional devitalized and mummified pulp. Its permeation of the tubuli makes its use advantageous when it is impracticable to thoroughly remove all of the semi-disorganized dentine. It arrests further destruction of that tissue and corrects the sensitiveness which renders further excavation impossible. Very sensitive cavities may be filled with no preparation beyond securing fairly good margins. After a few weeks they can be satisfactorily prepared for a more thorough operation. It is most satisfactory in stopping cavities in the temporary teeth, as it can be done after very indifferent preparation, which is a boon in the management of little patients, as the deciduous teeth can be preserved through their natural term by this means.

The fissures in the first permanent molars can be sealed up with this cement without putting the child to any discomfort, also in the treatment of those trying grooves and cavities in the buccal surfaces of molars and the sensitive place in the palatine surfaces caused by the contact of vulcanite or other plates. There is no necessity for anchorage in its use. Where there is a cavity below the gum-line with a fungous growth filling the cavity, excise the fungous growth and place a gutta-percha filling against it until the surface is healed, and then fill with cupric phosphate. The tissue will remain healthy in contact with the surface of this filling, as the cement presents a very smooth surface if not disturbed while crystallizing. It must be used in an almost fluid state; crystallization is hastened by projecting hot water upon the surface as it congeals. The moderate rubbing together of powder and fluid which suffices for the ordinary cements is not sufficient for this cupric phosphate. The powder is added directly to the liquid until a stiff mass is obtained; then with vigorous rubbing on a broad slab with a broad stiff spatula, the mass is reduced to a fairly watery state, the rubbing being continued till it is brought to an unctuous, creamy state, resembling oil-paint or printer's ink, which runs slowly from the spatula in a stringy manner. This confirms the statement that "the least reliable cements are the most easily manipulated." In packing, the cement will adhere to the cavity wall rather than to the packing instrument, though a film of the powder on the instrument will facilitate packing it. This cement has good working qualities and is very adhesive to dry tooth-surface. If the filling is not treated with hot water (protecting the soft tissues with napkins) a very objectionable copper-salt taste will be complained of.

Dr. W. G. Browne, Atlanta, Ga., read a brief paper entitled: "Varnishing Cavities."

Because of the admitted incompatibility of the metals and tooth structure, gutta-percha, chloro-percha, the cements and varnish have all been tried in the effort to overcome the difficulty. As a non-conductor against thermal changes and as an insulator against electrical changes, Dr. Browne finds that a clear resin, such as damar varnish, dissolved in chloroform, offers many advantages, as it is not soluble in the fluids of the mouth, does not discolor, and prevents the discoloration from the oxidation of amalgams showing through their enamel walls. It also acts as a support to frail walls by inserting the filling while the varnish is in a plastic state. For a large gold filling he utilizes the varnish to hold in place adherent to the dentine a mat of crystal gold, or the first cylinder, in commencing the filling. He wished it distinctly understood, however, that he did not depend upon the varnish to retain the filling itself but simply to facilitate the start, though with its assistance deep retaining pits are made unnecessary, slight undercuts being all that is required, thus saving valuable time. No harm can possibly result from its use, while its advantages are clearly apparent.

Dr. W. H. Morrison, St. Louis, Mo., read a brief paper on "The Care of Infant's Teeth."

He said that, as a rule, the dentist is not consulted early enough, parents waiting till the teeth are eroded and decayed beyond repair. When they are placed in our charge from infancy, we should adopt the line of treatment suggested by nature, when the child stuffs its fists into its mouth, biting on anything within reach. This is very suggestive and if the mother or nurse will systematically rub the alveolar ridge and palate with the thumb and fingers, expanding the arch in the natural direction this will greatly aid their growth and development. The lancet should also be used over the points of greatest tension. This alone will often bring the child out of spasms and save its life. Do not give drugs and "soothing syrups," but rely on physical development. If a change has to be made from mother's milk, then give the milk from a healthy field-fed cow. Condensed-milk-teeth are not up to the standard. Rely on outdoor air and sunlight, physical culture and development of all parts of the body harmoniously, and in troubled dentition adopt this system of massage in the mouth. This is also to be recommended for older children in cutting the second teeth. By pressure of the thumb and fingers or the knuckles on too prominent points inside or outside of the arch will aid greatly in preventing irregularities, as the parts are cartilaginous and yield readily to such pressure.

These papers were passed without discussion.

[*To be continued.*]

